# Plasma interleukin-17 and alpha-fetoprotein combination effectively predicts imminent hepatocellular carcinoma occurrence in liver cirrhotic patients

**DOI:** 10.1186/s12876-021-01761-1

**Published:** 2021-04-17

**Authors:** Kung-Hao Liang, Ming-Wei Lai, Yang-Hsiang Lin, Yu-De Chu, Chih-Lang Lin, Wey-Ran Lin, Ya-Hui Huang, Tong-Hung Wang, Rong-Nan Chien, Tsung-Hui Hu, Chau-Ting Yeh

**Affiliations:** 1 Department of Medical Research, Taipei Veterans General Hospital, Taipei, Taiwan; 2 Institute of Food Safety and Health Risk Assessment, National Yang Ming Chiao Tung University, Taipei, Taiwan; 3 Institute of Biomedical Informatics, National Yang Ming Chiao Tung University, Taipei, Taiwan; 4 Liver Research Center, Chang Gung Memorial Hospital, Linkou, Taiwan; 5 Liver Research Unit, Keelung Chang Gung Memorial Hospital, No. 222, Maijin Road, Keelung, Taiwan; 6 Community Medicine Research Center, Keelung Chang Gung Memorial Hospital, Keelung, Taiwan; 7 Tissue Bank, Chang Gung Memorial Hospital, Tao-Yuan, Taiwan; 8 Division of Hepatogastroenterology, Department of Internal Medicine, Kaohsiung Chang Gung Memorial Hospital, Kaohsiung, Taiwan; 9 Molecular Medicine Research Center, Chang Gung University, Taoyuan, Taiwan; 10 Liver Research Center, Chang Gung Memorial Hospital, 5, Fu-Shin street, Kuei-Shan District, Taoyuan, Taiwan

**Keywords:** Immunology, Inflammation, Th17, Oncogenesis

## Abstract

**Background:**

Predicting imminent hepatocellular carcinoma (HCC) in liver cirrhotic patients is an unmet medical need. We aimed to investigate circulatory biomarkers and their optimum combinations in a prospective study.

**Methods:**

We investigated plasma interleukin 17 (IL-17) concentrations, quantified using enzyme-linked immunosorbent assay (ELISA), for the prediction of HCC in a large cohort of 404 HCC-naïve liver cirrhotic patients regularly followed after recruitment. Additionally, IL-17 in surgically resected tumor tissues were evaluated using immunohistochemistry staining.

**Results:**

IL-17 was detected in HCC tissues. The IL-17 concentrations in the peripheral blood do not have correlation with an extensive list of 31 common demographic, metabolic and liver function variables in the cohort of liver cirrhotic patients. Furthermore, patients stratified by IL-17 and alpha-fetoprotein (AFP) showed distinctive cumulative incidence of HCC. Imminent HCC, defined here as HCC occurrence within 1 year, can be predicted by IL-17 alone with an area under the receiver operating characteristic curve [AUC] of 0.762 (*P* = 0.002). An multivariate analysis showed that age, hepatitis C viral infection, AFP and IL-17 were four independent factors associated with imminent HCC (adjusted *P* = 0.03, 0.041, 0.024 and 0.008 respectively). An explicit risk score (R) combining the concentrations of two plasma biomarkers, AFP and IL-17, achieved a high AUC of 0.933 (95% confidence interval 0.893–0.972, *P* < 0.001) in predicting imminent HCC, with 100% sensitivity and 79.9% specificity at the optimum cutoff. The score is defined as: ${\text{R}} = (2.6914)*{\text{IL-17}} + (0.3909)*{\text{AFP}} - (0.80812875)*{\text{IL-17}}^{2} + (0.10288876884)*{\text{IL-17}}^{2} *{\text{AFP}}.$

**Conclusions:**

The circulatory IL-17 concentration is a predictor of subsequent HCC occurrence in liver cirrhotic patients. The combination of AFP and IL-17 is highly effective in predicting imminent HCC within 1 year.

**Supplementary Information:**

The online version contains supplementary material available at 10.1186/s12876-021-01761-1.

## Background

Liver cirrhosis is a life-threatening disease responsible for more than 1,000,000 deaths annually worldwide [1]. Persistent or intermittent liver inflammation due to viral infection, alcohol toxicity, nonalcoholic steatohepatitis or autoimmune hepatitis can cause liver fibrosis and cirrhosis. One major sequela of liver cirrhosis is hepatocellular carcinoma (HCC), occurring at an annual rate of 2–5% [2]. Knowing exactly when HCC will occur is highly pertinent to the clinical care of cirrhotic patients. However, it seems unpredictable for individual patients. In some patients, HCC develops within 1 year, while in others, HCC never occurs in the patient’s lifetime. In the past, noninvasive scores such as fibrosis-4 (FIB-4), AST to Platelet Ratio Index (APRI) and gamma-glutamyl transpeptidase-to-platelet ratio have been developed to estimate the severity of fibrosis [3, 4]. These scores were subsequently used for the prediction of HCC [5–11], based on the rationale that the severity of fibrosis correlates with HCC risks. Noninvasive liver stiffness measurements were also used for the prediction of HCC [12] and posttreatment prognosis [13]. The organic anion transporter peptides (OATP) 1B1 and 1B3 has also been shown to be indicative of HCC recurrence after liver transplanation [14].

Alpha-fetoprotein (AFP) is a diagnostic biomarker of HCC and one of the most studied predictive biomarkers of imminent HCC that has high specificity [15–21]. However, HCC can still occur in patients without prominent AFP elevation, resulting in unsatisfactory sensitivity using AFP alone [15]. AFP-L3 and proteins induced by vitamin K absence (PIVKA-II) are two other biomarkers with clinical potential for HCC screening. However, the two biomarkers are rarely studied and used outside of Japan [22]. Thus, biomarkers and their optimum combinations for the prediction of subsequent HCC in liver cirrhotic patients remain an unmet medical need.

Recent pilot studies have unveiled the involvement of a proinflammatory cytokine, interleukin-17 (IL-17), in the pathogenesis of liver fibrosis, cirrhosis and autoimmune hepatitis. IL-17 is frequently elevated in patients with liver cirrhosis [23–26], autoimmune hepatitis [27], steatohepatitis [28, 29], and alcohol-related HCC [30]. Circulating and tissue-infiltrating Th17 cells were reported to correlate with the severity of liver inflammation in chronic hepatitis C patients [31]. Serum IL-17 levels were also observed to be in correlation with alanine aminotransferase (ALT) in an aggregated cohort of chronic hepatitis B patients and healthy controls [32], implying that ALT and IL-17 levels are both high in chronic hepatitis B patients and low in healthy controls. Another study showed that serum IL-17 was higher in cirrhotic hepatitis B patients than in asymptomatic hepatitis B carriers [33]. The fibrosis stages of patients were also positively correlated with IL-17 levels in the serum and liver [33]. A higher proportion of circulating inflammatory cells was observed in patients with higher IL-17 levels, in whom the histology activity index was also higher [34].

IL-17 has also been observed in other pilot studies on the prognosis of HCC. The serum levels of IL-17 in HCC patients receiving surgical resections are indicative of poor time to progression and overall survival [35] as well as higher subsequent recurrence rates [36]. The intratumoral levels of IL-17 and its receptor are also associated with poorer postresection HCC recurrence and patient survival [35, 37]. Apart from HCC, IL-17 has been reported to be involved in other solid cancers such as non-small cell lung cancer [38] and medulloblastoma [39], suggesting its broad involvement in oncogenesis.

These pilot studies motivated us to investigate whether circulatory IL-17 concentrations could serve as a clinically useful biomarker for predicting HCC occurrence. Hence, we recruited a homogeneously cirrhotic, HCC-naïve patient cohort with extensive assessments of clinical variables, including plasma IL-17 concentrations and AFP. The patients were regularly followed after recruitment to monitor the occurrence of HCC at the earliest time possible.

## Methods

### Patients

This study was approved by Chang Gung Memorial Hospital, Taiwan, and conducted according to the 1975 Declaration of Helsinki. A total of 404 HCC-naïve, adult liver cirrhotic patients were recruited from three branches of Chang Gung Memorial Hospital in northern, central-northern and southern Taiwan. Patients with liver decompensation were excluded. All the patients provided informed consent. Cirrhosis was diagnosed by either (1) liver biopsy, or (2) ultrasound imaging in conjunction with endoscopy or transient elastography (FibroScan; Echosens, France). Basic demographic variables, viral etiology and general blood biochemistry measurements were documented at the time of patient recruitment (c.f. Table 1 for a complete list). This study was performed between 2013/1/1 and 2017/11/8. During this period, the patients were regularly followed every 3 months to monitor HCC occurrence. Chronic hepatitis B patients receive antiviral treatments if their HBV DNA levels were greater than 2000 IU/mL. The recruited HCV patients were not treated initially until direct-acting antivirals (DAAs) were covered by national health insurance in Taiwan in 2017. Hepatocellular carcinoma was diagnosed by: (i) echo-guided liver biopsy or fine-needle aspiration cytology, (ii) AFP > 200 ng/mL, tumor > 2 cm and typical HCC characteristics in dynamic computed tomography, or (iii) typical HCC characteristics in both dynamic computed tomography and angiography. All HCC patients diagnosed during this study were at Barcelona Clinic Liver Cancer stage A.

**Table 1:** Basic clinical and serum biochemistry variables and their association with circulatory IL-17 concentrations

Item	Values	Beta	Confidence interval		*P*
		Coefficient	Low	High	
Patient #	404				
IL-17 (ng/ml)	5.57 ± 18.74				
Age (years)	59.37 ± 10.94	91.4	− 76.4	259.2	0.285
Gender-Male	249 (61.6%)	1814.3	− 1956.2	5584.8	0.345
HBV positivity^†^	237 (58.7%)	− 1293.9	− 5019.2	2431.4	0.495
HCV positivity^‡^	128 (31.7%)	2587.2	− 1350.0	6524.4	0.197
AST (IU/L)	45.38 ± 40.61	− 2.9	− 48.2	42.5	0.900
ALT (IU/L)	39.76 ± 45.48	− 13.7	− 54.2	26.8	0.506
Bilirubin (mg/dL)	1.43 ± 5.47	− 7.4	− 344.1	329.3	0.965
AFP (ng/ml)	8.84 ± 28.99	21.6	− 41.9	85.0	0.505
Albumin (g/dL)	3.61 ± 0.44	1222.5	− 3006.3	5451.2	0.570
Total protein (g/dL)	7.21 ± 0.67	− 455.5	− 3188.5	2277.5	0.743
C3 (mg/dL)	96.42 ± 20.62	− 73.7	− 163.1	15.8	0.106
C4 (mg/dL)	19.68 ± 8.40	− 77.7	− 297.9	142.5	0.488
TSH (mU/L)	2.16 ± 4.70	29.0	− 360.5	418.5	0.884
Free T4 (ng/dL)	1.07 ± 0.26	− 2422.4	− 9503.0	4658.2	0.502
Sugar AC (mg/dL)	106.81 ± 35.66	− 14.1	− 65.8	37.7	0.593
Glycohemoglobin (%)	6.07 ± 1.33	− 447.7	− 1838.8	943.5	0.527
Insulin (mIU/L)	11.33 ± 12.31	− 103.3	− 252.6	46.1	0.175
Insulin/Sugar	0.38 ± 2.21	− 222.5	− 1128.2	683.2	0.629
HOMA-IR	2.68 ± 3.19	− 243.9	− 868.3	380.4	0.442
Apo A1 (g/L)	1.37 ± 0.30	− 4431.4	− 10,512.7	1649.8	0.153
Uric Acid (mg/dL)	5.97 ± 1.79	− 695.8	− 1730.0	338.5	0.187
HDL (mg/dL)	48.70 ± 16.27	15.9	− 97.3	129.2	0.782
VLDL (mg/dL)	20.67 ± 13.88	− 40.1	− 175.2	95.0	0.560
LDL (mg/dL)	97.35 ± 30.80	− 30.7	− 91.3	29.8	0.319
Cholesterol (mg/dL)	165.81 ± 36.85	− 26.5	− 76.8	23.8	0.302
Triglyceride (mg/dL)	104.20 ± 63.69	− 8.5	− 37.7	20.6	0.565
Ferritin (ng/mL)	274.01 ± 445.64	1.0	− 3.2	5.1	0.651
Ceruloplasmin (mg/dL)	24.79 ± 5.43	15.1	− 327.7	358.0	0.931
Iron (ug/dL)	119.29 ± 55.86	24.9	− 8.1	57.8	0.139
TIBC (ug/dL)	326.69 ± 56.80	− 13.2	− 45.7	19.3	0.424
UIBC (ug/dL)	200.22 ± 84.92	− 14.3	− 42.0	13.3	0.309

^†^“HBV positivity” include (a) HBV without HCV, n = 219; and (b) HBV, HCV co-infection, n = 18

^‡^“HCV positivity” include (a) HCV without HBV, n = 110; and (b) HBV, HCV co-infection, n = 18

Additionally, the surgically resected tumor tissue samples of 4 early-stage HCC patients were evaluated. The flowchart of this study is presented in Additional file 1: Figure S1.

### Quantifying circulatory IL-17 concentrations

Peripheral blood samples were collected at patient recruitment, centrifuged and then stored in − 20 °C for the subsequent quantification of IL-17 concentrations using the Human IL-17A/F Heterodimer DuoSet Enzyme-Linked Immunosorbent Assay (ELISA) Kit (DY5194-05), which was purchased from R&D systems (Minneapolis, MN, USA).

### Immunohistochemistry staining of IL-17 and AFP in resected HCC tissues

Immunohistochemistry staining of IL-17 and AFP was performed on surgically resected HCC tissues preserved in the formalin-fixed paraffin-embedded (FFPE) blocks and cut into sections with a thickness of 5 μm. The FFPE samples were deparaffinized, dehydrated and incubated with the appropriate antibodies (ab136668 and ab46799, Abcam, Cambridge, UK) for the staining.

## Results

### IL-17 and AFP are present in HCC tissues

We first evaluated whether IL-17 and AFP can be found in the tumor tissues of patients in Taiwan. We performed immunohistochemical (IHC) staining of IL-17 and AFP in surgically resected tumor tissues of 4 patients, and discovered that IL-17 and AFP were not homogeneously distributed in all patients we evaluated; rather, tissue of some patients showed signal of IL-17 but not AFP, while others showed signal of AFP but not IL-17. Images of such IHC results are presented in Additional file 1: Figure S2.

### Circulatory IL-17 concentration has no correlation with 31 demographic, metabolic and liver function variables

The baseline clinical variables of the patient cohort are summarized in Table 1. We first investigated the correlations between circulatory IL-17 concentrations and an extensive list of 31 baseline demographic variables and general biochemistry measurements pertaining to liver functions and metabolic disorders. IL-17 did not show a correlation with any of these variables (Table 1).

### Circulatory IL-17 is indicative of subsequent HCC at several cutoff points

**Fig. 1:**
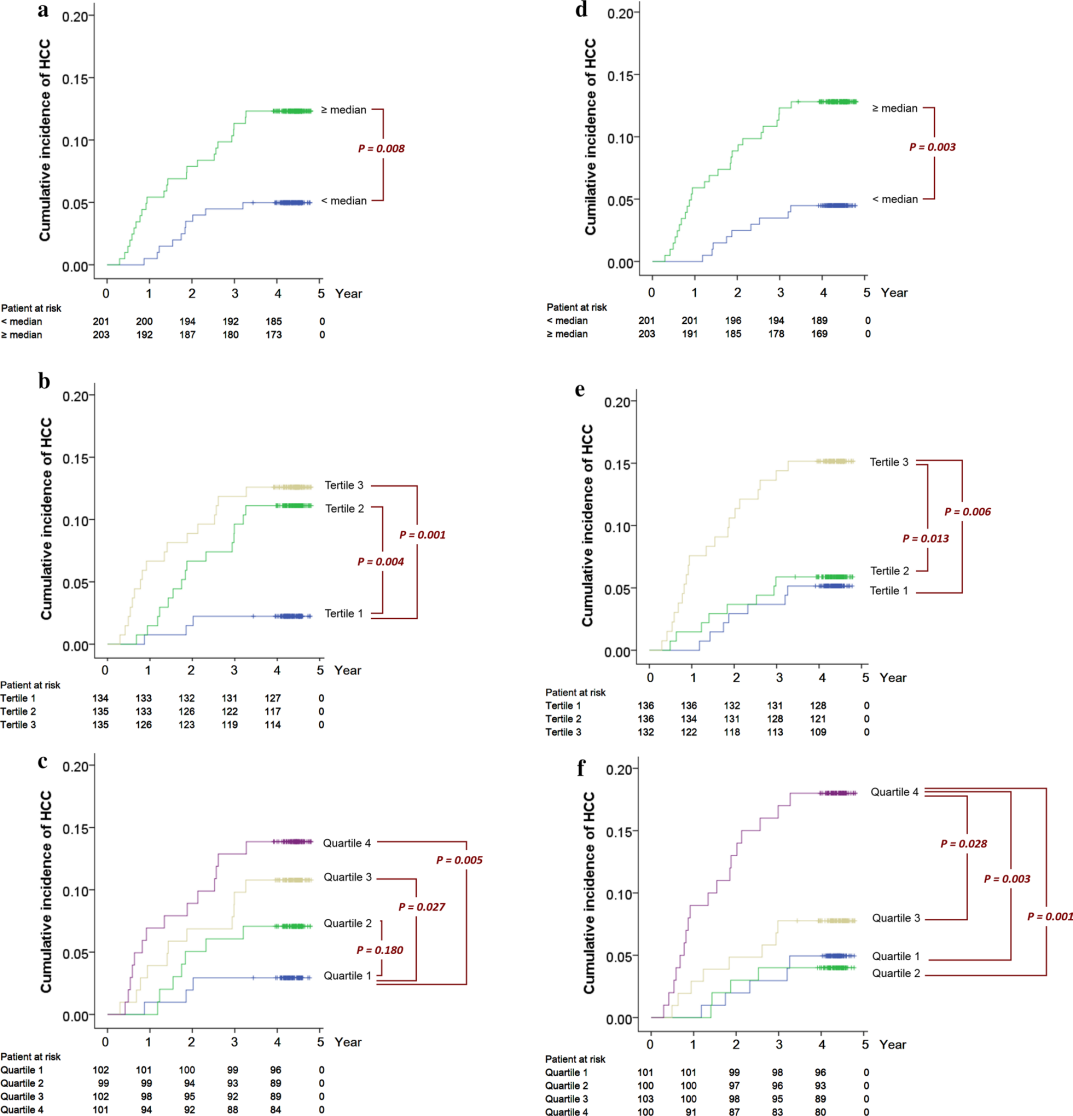
Evaluation of patient strata with respect to their subsequent HCC development using the Kaplan–Meier visualizations. The patients were stratified by IL-17 concentrations into **a** two equal sizes above and below the median; **b** tertiles; and **c** quartiles. Similarly, patients were stratified by AFP concentrations (**d**–**f**). Comparisons with *P* > 0.05 are not labeled in this figure for simplification

A preferable characteristic of a clinical biomarker is that it would remain effective in discriminating patients with different risk levels or disease severity levels in a wide range of cutoff points. We therefore evaluated IL-17 at various cutoff points with Kaplan–Meier visualization. The patients were stratified into 2, 3 and 4 equal-sized groups based on their plasma IL-17 concentrations. When the median value (455 pg/ml) was used as the cutoff point, patients with IL-17 ≥ the median had a significantly higher cumulative incidence of HCC than those with IL-17 < the median (*P* = 0.008, Fig. 1a). When the patients were divided into tertiles (using the cutting values of 140 and 1105 pg/ml), the cumulative incidence of tertile 1 (the group with the lowest IL-17 levels) was consistently lower than that of tertile 2 and tertile 3 (log-rank *P* = 0.004 and 0.001 respectively, Fig. 1b). Patients in tertile 2 and tertile 3 showed non-overlapping Kaplan–Meier curves, yet the difference did not reach statistical significance (*P* = 0.653). When the patients were divided into quartiles (using cutoff values of 20, 455 and 1850 pg/ml), patients in quartile 1 ~ 4 showed four visually distinct curves, ordered from bottom to top in Fig. 1c. The cumulative HCC incidence of quartile 1 was significantly lower than that of quartile 3 and quartile 4 (*P* = 0.027 and 0.005 respectively). Pairwise comparisons between the other strata do not show statistical significance, despite the visually distinct curves (Quartile 1 vs. 2 *P* = 0.180; Quartile 2 vs. 4 *P* = 0.112; Quartile 2 vs. 3 *P* = 0.353; Quartile 3 vs. 4 *P* = 0.485). The cumulative incidences of the patient strata are shown in Table 2. The risk of HCC was relatively proportional to the IL-17 levels, facilitating the use of different cutoffs for different clinical purposes (Fig. 1a–c).

**Table 2:** Cumulative incidence of HCC in different patient groups stratified by IL-17 and AFP concentrations

	IL-17					AFP				
	Cutoff	Cumulative incidence at year				Cutoff	Cumulative incidence at year			
	(pg/ml)	1	2	3	4	(ng/ml)	1	2	3	4
< Median	< 455	0.000	0.035	0.045	0.050	< 3.9	0.000	0.025	0.035	0.045
≥ Median	≥ 455	0.059	0.079	0.113	0.126	≥ 3.9	0.059	0.089	0.123	0.128
Tertile 1	< 140	0.000	0.015	0.022	0.022	< 3	0.000	0.029	0.037	0.051
Tertile 2	< 1105	0.015	0.067	0.096	0.111	< 4.9	0.015	0.037	0.059	0.059
Tertile 3	≥ 1105	0.076	0.089	0.119	0.126	≥ 4.9	0.076	0.106	0.144	0.152
Quartile 1	< 20	0.000	0.020	0.029	0.029	< 2.7	0.000	0.020	0.030	0.050
Quartile 2	< 455	0.000	0.051	0.061	0.071	< 3.9	0.000	0.030	0.040	0.040
Quartile 3	< 1850	0.029	0.069	0.098	0.108	< 6	0.029	0.049	0.078	0.078
Quartile 4	≥ 1850	0.090	0.089	0.129	0.143	≥ 6	0.090	0.130	0.170	0.180

We also analyzed patient strata by AFP concentrations. When the patents were divided by the median (3.9 ng/ml), those with higher AFP had a significantly higher cumulative incidence of HCC (*P* = 0.003, Fig. 1d). When the patients were stratified into tertiles by the cutoffs of 3.0 and 4.9 ng/ml, the cumulative incidence of tertile 1 and tertile 2 was consistently lower than that of tertile 3 (*P* = 0.006 and 0.013 respectively, Fig. 1e). When the patients were stratified into quartiles by the cutoffs of 2.7, 3.9 and 6.0 ng/ml, the significance levels of difference in cumulative HCC incidence between quartiles 1 and 4, 2 and 4, and 3 and 4 were 0.003, 0.001 and 0.028 respectively (Fig. 1f). Pairwise comparisons between the other strata do not show statistical significance. The curves of quartile 1 and quartile 2 are intertwined.

**Fig. 2:**
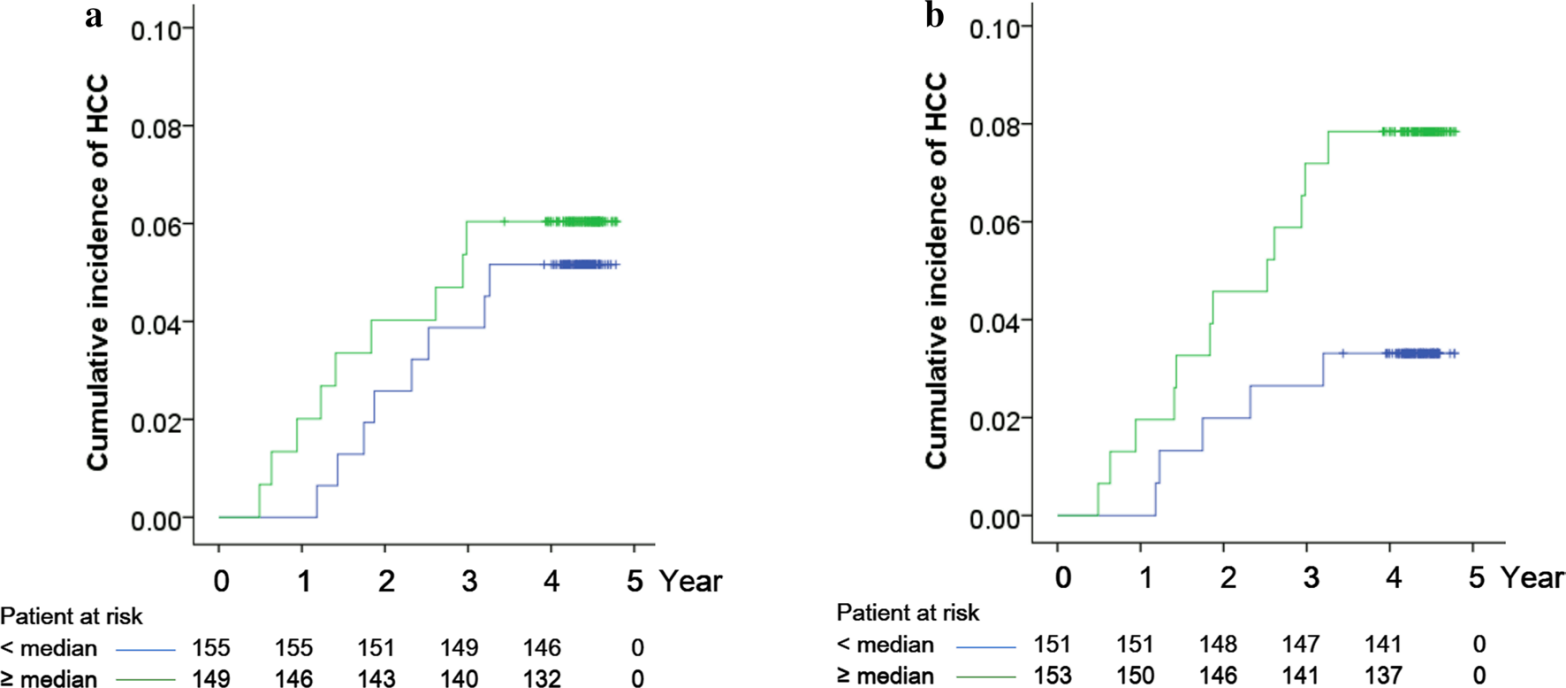
The cumulative incidence of HCC in patients with AF *P* < 6.0 ng/ml. **a** Patients were stratified by the median of AFP (*P* = 0.723). The cumulative incidence of HCC at year 4 of patients with AF *P* < median and ≧ median are 0.05 and 0.06 respectively. **b** Patients were stratified by the median of IL-17 (*P* = 0.086). The cumulative incidence of HCC at year 4 of patients with IL-17 < median and ≧ median are 0.03 and 0.08 respectively

Figure 1f and Table 2 show that AFP is useful for indicating a subset of high-risk patients whose AFP ≧ 6.0 ng/ml, corresponding to the 4^th^ quartile. We further asked that once these patients have been identified as high-risk patients due to their high AFP levels, how can the remaining patients be further stratified to reflect their different risk levels? In patients with AF *P* < 6.0 ng/ml, the cumulative incidences of HCC at year 4 of patients IL-17 < median and ≧ median are 0.03 and 0.08 respectively (Fig. 2b), suggesting that IL-17 levels can reflect the different HCC incidence in patients with low AFP. In contrast, the cumulative incidence of HCC at year 4 of patients with AF *P* < median and ≧ median are 0.05 and 0.06 respectively (Fig. 2a), which are relatively close to each other.

### IL-17 concentrations were particularly effective in indicating HCC at one year

**Fig. 3:**
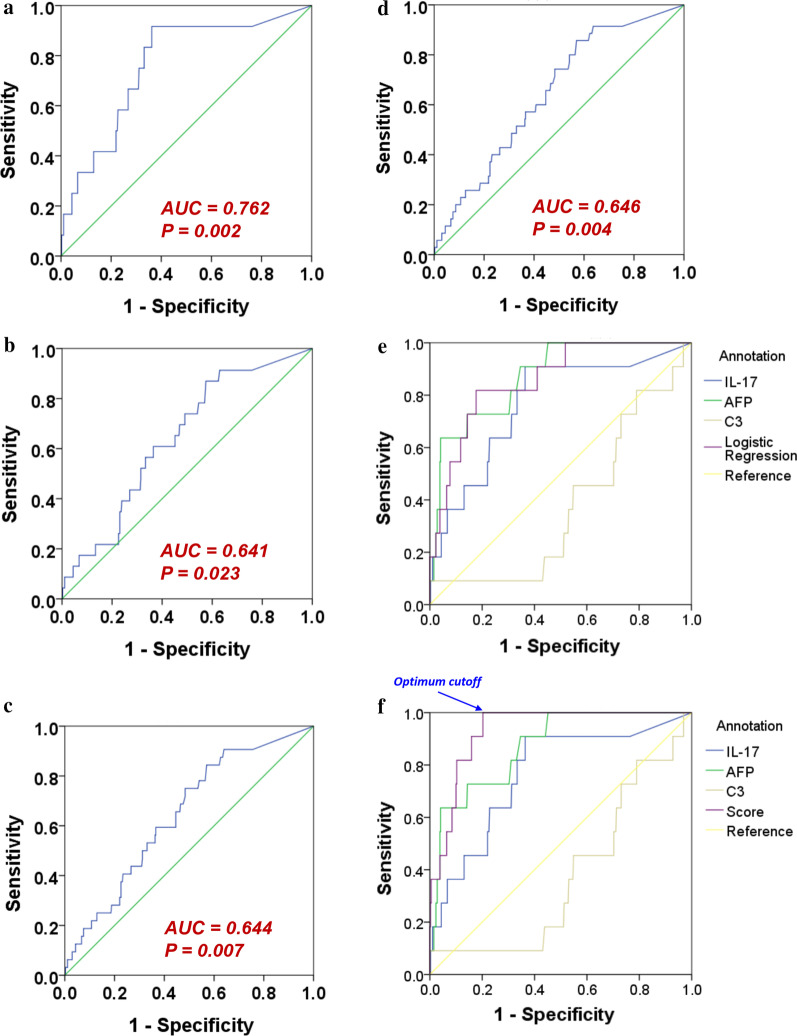
The performance of biomarkers and their combinations for predicting the occurrence of HCC, shown by receiver operating characteristic curves. **a** The prediction by IL-17 alone of HCC events at 1 year; **b** 2 years; **c** 3 years (n = 404) and **d** 4 years (n = 393; 11 cirrhosis patients were censored in year 4). **e** The performance of predicting HCC events at 1 year using IL-17, AFP, C3 and the multivariate logistic regression Eq. (1). **f** The prediction of HCC events at 1 year using IL-17, AFP, C3 and the risk score combining IL-17 and AFP defined in Eq. (2)

We then analyzed the occurrence of HCC at different time points, including 1-, 2- 3- and 4-years after peripheral blood was drawn from the patients. It was found that the IL-17 concentrations could significantly classify patients with or without HCC at all these time points (Fig. 3a–d, *P* = 0.002, 0.023, 0.007 and 0.004 for 1–4 years respectively). Although the data suggest that IL-17 can reflect HCC occurrence in the next several years after baseline, the best performance was at year 1, with the highest area under the receiver operating characteristic curve (AUC = 0.762) and the lowest *P*-value (0.002). The results indicate that circulatory IL-17 concentrations were particularly effective for predicting imminent HCC, defined as the HCC occurrence within 1 year of sample collection.

### Investigating the synergistic effect of IL-17 and other clinical variables

We then evaluated IL-17 and an extensive list of variables including viral etiology, gender (male, female), AFP, and general metabolic and liver function variables, for their association with imminent HCC using univariate logistic regression. Four variables, IL-17, age, HCV and AFP, were indicative of HCC (all *P* < 0.05, Table 3). When the four factors were analyzed by multivariate logistic regression, all of them remained statistically significant. This finding showed that IL-17 (adjusted *P* = 0.008, Table 3) and the other three factors are independent predictors of imminent HCC.

**Table 3:** Univariate and multivariate analysis of variables associated with imminent HCC

Item	Odds ratio	Confidence interval		*P*
		Low	High	
*Univariate analysis*				
IL-17 (pg/ml)	1	1	1	**0.004**
Age (years)	1.079	1.017	1.145	**0.012**
Gender-Male	0.613	0.194	1.936	0.404
HBV	0.341	0.101	1.152	0.083
HCV	6.882	1.831	25.874	**0.004**
AST (IU/L)	1.003	0.995	1.012	0.443
ALT (IU/L)	1.003	0.996	1.01	0.343
Bilirubin (mg/dL)	0.89	0.369	2.151	0.797
AFP (ng/ml)	1.014	1.004	1.024	**0.005**
Albumin (g/dL)	0.476	0.17	1.332	0.157
Total protein (g/dL)	0.834	0.401	1.735	0.628
C3 (mg/dL)	0.989	0.96	1.018	0.435
C4 (mg/dL)	0.949	0.867	1.037	0.248
TSH (mU/L)	0.448	0.179	1.121	0.086
Free T4 (ng/dL)	0.099	0.003	3.519	0.205
Sugar AC (mg/dL)	1.004	0.99	1.018	0.578
Glycohemoglobin (%)	1.206	0.864	1.684	0.270
Insulin (mIU/L)	1.014	0.981	1.048	0.409
Insulin/sugar	0.425	0.001	305.942	0.799
HOMA-IR	1.1	0.976	1.241	0.118
Apo A1 (g/L)	2.573	0.811	8.165	0.109
Uric acid (mg/dL)	0.855	0.588	1.243	0.412
HDL (mg/dL)	0.977	0.935	1.02	0.285
VLDL (mg/dL)	1.011	0.978	1.045	0.516
LDL (mg/dL)	0.983	0.961	1.005	0.132
Cholesterol (mg/dL)	0.993	0.977	1.008	0.346
Triglyceride (mg/dL)	1.003	0.995	1.01	0.525
NonHDLC	0.991	0.971	1.011	0.368
Ferritin (ng/mL)	1	0.999	1.001	0.768
Ceruloplasmin (mg/dL)	1.029	0.926	1.145	0.593
Iron (ug/dL)	1.005	0.996	1.014	0.263
TIBC (ug/dL)	1.001	0.99	1.011	0.891
UIBC (ug/dL)	0.997	0.988	1.007	0.591
*Multivariate analysis*				
IL-17 (pg/ml)	1	1	1	**0.008**
Age (years)	1.081	1.008	1.161	**0.030**
HCV	4.257	1.059	17.113	**0.041**
AFP	1.012	1.002	1.023	**0.024**

*P* values smaller than 0.05 are shown in bold

The coefficients of the four variables in the multivariate logistic regression analysis offered the following combination formula:

1 $$0.021*{\text{IL-17}} + 0.078*{\text{age}} + 1.449*{\text{HCV}} + 0.012*{\text{AFP}}$$

The synergistic effect determined by the above formula (AUC = 0.866), however, did not have a large-enough improvement with statistical significance from the predictive effect of IL-17 or AFP alone (*P* = 0.163 and 0.445, respectively, Fig. 3e).

We further evaluated the best possible performance for combining IL-17 and AFP. This time, we used an algorithm that we had developed in house, the generalized iterative modeling method (GIM), to find the optimum polynomial combination of biomarkers in terms of AUC [40]. The source code of GIM can be found on the following public-domain GitHub website: https://github.com/khliang/GIM.

A risk score (R) was thus developed using this algorithm.

2 $${\text{R}} = 2.6914*{\text{IL-17}} + (0.3909)*{\text{AFP}} - (0.80812875)*{\text{IL-17}}^{2} + (0.10288876884)*{\text{IL-17}}^{2} *{\text{AFP}}$$

The AUC of this risk score was 0.933 (confidence interval = [0.893–0.972], Fig. 3f). It had a significantly better performance than IL-17 alone (AUC = 0.762, *P* = 0.037). The performance was also better than that of AFP alone (AUC = 0.871) but did not achieve statistical significance (*P* = 0.255). The optimum cutoff, based on Youden’s index [41], occurred at a score of 4.5072 (Fig. 3f). At this cutoff, the sensitivity and specificity were 100% and 79.9% respectively. We also applied this score to patient subsets of the two major etiologies: chronic hepatitis B (n = 237) and chronic hepatitis C (n = 128). The AUC were 0.950 [0.894–1] and 0.894 [0.825–0.964] for chronic hepatitis B and C patients respectively (both *P* < 0.001).

## Discussion

Liver fibrosis is a progressive disease which usually persists for decades and has multiple stages according to the definition by the Ishak or METAVIR systems [42]. Previously, non-invasive APRI and FIB-4 scores have been developed to indicate fibrosis stages, which naturally correlated with the HCC risks. More advanced stages correspond to higher HCC risks [5–11]. The current study addressed liver cirrhotic patients, corresponding to those in the stages 5 and 6 of the Ishak fibrosis staging system and stage 4 in the METAVIR staging system. HCC risks of liver cirrhotic patients have increased to a degree that imminent HCC is one major concern of patient care. Hence, additional biomarkers is required so as to provide high sensitivity and specificity of predicting imminent HCC in liver cirrhotic patients.

To date, ALT and AFP have been widely used for the assessment of liver disorders. ALT is released from the liver to the blood whenever there is liver damage. Thus, the circulatory levels of ALT are routinely used to indicate the extent of liver damage, acute liver inflammation and hepatitis [4, 43]. AFP is an onco-fetoprotein produced by cancer cells. A wealth of literature has shown the usefulness of AFP for indicating imminent HCC [15–21]. In addition to the two biomarkers (ALT and AFP) attributed to hepatitis flares, IL-17 represents a distinct immunological aspect of the disease. IL-17 is a major cytokine mediating the Th-17 response. Based on recent pilot studies showing the correlation of high IL-17 levels with various liver-related clinical endpoints [28, 30, 33, 35, 37, 44, 45], we performed a prospective cohort study, which showed that the circulatory IL-17 concentration can serve as a clinical biomarker for predicting imminent HCC. We showed that IL-17 can predict imminent HCC effectively, independent of age, viral etiology (i.e., HCV) and AFP.

We explored two different methods of utilizing both AFP and IL-17, two distinct aspects of HCC biology. First, we evaluated a hierarchical patient stratification. Patients with AFP ≧ 6.0 ng/ml are identified as at high risk of HCC. The remaining patients were further stratified by the medians of AFP and IL-17 subsequently to evaluate the performance of these two biomarkers. The cumulative incidence of the two patient strata by AFP are pretty close to each other, suggesting an unsatisfactory stratification (Fig. 2a). In contrast, when these patients were stratified by IL-17, those with higher IL-17 level have higher cumulative incidence of HCC (Fig. 2b), demonstrating the usefulness of IL-17 as the second biomarker in the hierarchical patient stratification. Second, we evaluated a polynomial model of predicting imminent HCC using AFP and IL-17. This model achieved a high area under the ROC of 0.933 in our patient cohort.

The predictability of IL-17 might be partly due to recently elucidated oncogenic mechanisms. IL-17 has been found to interact with EGFR, FGFR, NOTCH1 and C-type lectin signaling [38, 45, 46]. The oncogenic mechanisms of the IL-17 driven, IL-17/STAT3/Notch1 [38] and IL-17/STEAP4/XIAP axes have been proposed, where IL-17 drives the intake of copper alongside the metalloreductase STEAP4 [47]. These mechanisms have been elucidated in solid cancers other than hepatocellular carcinoma. Thus, the exact oncogenic mechanism of IL-17 in HCC remains the goal of future investigations.

In this cirrhotic cohort, we observed that IL-17 concentrations did not correlate with an extensive list of 31 common clinical variables including AFP and ALT (Table 1). Correlation is a statistical method widely used for clinical studies. Correlation does not indicate causation, however, causation may manifest as numerical correlation between variables which offered clues for further investigations. Since there is no correlation found, no straightforward causation can be inferred between IL-17 and the studied variables.

Motivated by literature, we chose IL-17 as the main target of evaluation. This study is limited by the fact that no other cytokines were evaluated. Better biomarkers may be found among other cytokines which remained to be explored in future studies. Also, the current study is performed in patients in Taiwan. More studies are required to extrapolate the results into other populations in more geographical regions.

## Conclusion

The circulatory IL-17 concentration is indicative of imminent HCC in liver cirrhotic patients. The combination of AFP and IL-17 can predict imminent HCC with a high AUC of 0.933.

## Supplementary Information

**Additional file 1: Supplementary Figure 1**. The flow chart of this study. **Supplementary Figure 2**. The immunohistochemistry staining of IL-17 and AFP in tumor tissues.

## Data Availability

The datasets of this study are available from the corresponding authors on reasonable requests.

## Abbreviations

HCC: Hepatocellular carcinoma
FIB-4: Fibrosis-4
APRI: AST to Platelet Ratio Index
OATP: Organic anion transporter peptides
HDL: High density lipoprotein
HBV: Hepatitis B virus
HCV: Hepatitis C virus
AST: Aspartate aminotransferase
ALT: Alanine transaminase
AFP: Alpha-fetoprotein
AC: Ante cibum
T4: Thyroxin
Apo-A1: Apolipoprotein-A1
UA: Uric acid
LDL: Low density lipoprotein
VLDL: Very low density lipoprotein
TIBC: Total iron binding capacity
UIBC: Unsaturated iron binding capacity
C3: Complement component 3
C4: Complement component 4
BUN: Blood urea nitrogen
HOMA-IR: Homeostasis model assessment-estimated insulin resistance
PT: Prothrombin time
FFPE: Formalin-fixed paraffin-embedded
AUC: Area under the receiver operating characteristic curves
GIM: Generalized iterative modeling
