# Supplementary material for: Plasma interleukin-17 and alpha-fetoprotein combination effectively predicts imminent hepatocellular carcinoma occurrence in liver cirrhotic patients
Source: BMC Gastroenterol. 2021 Apr 17;21:177. doi: 10.1186/s12876-021-01761-1 (PMC8052794; doi:10.1186/s12876-021-01761-1)
Supplement: Supplementary file 1 — Additional file 1: Supplementary Figure 1. The flow chart of this study. Supplementary Figure 2. The immunohistochemistry staining of IL-17 and AFP in tumor tissues. [file 12876_2021_1761_MOESM1_ESM.pdf]

Plasma interleukin-17 and alpha-fetoprotein combination effectively predicts imminent hepatocellular carcinoma occurrence in liver cirrhotic patients

Kung-Hao Liang[1,2,3,*], Ming-Wei Lai[4], Yang-Hsiang Lin[4], Yu-De Chu[4], Chih-Lang Lin[4,5,6], Wey-Ran Lin[4], Ya-Hui Huang[4], Tong-Hung Wang[4,7], Rong-Nan Chien[4,5], Tsung-Hui Hu[8], Chau-Ting Yeh[4,9,*]

Supplementary Information

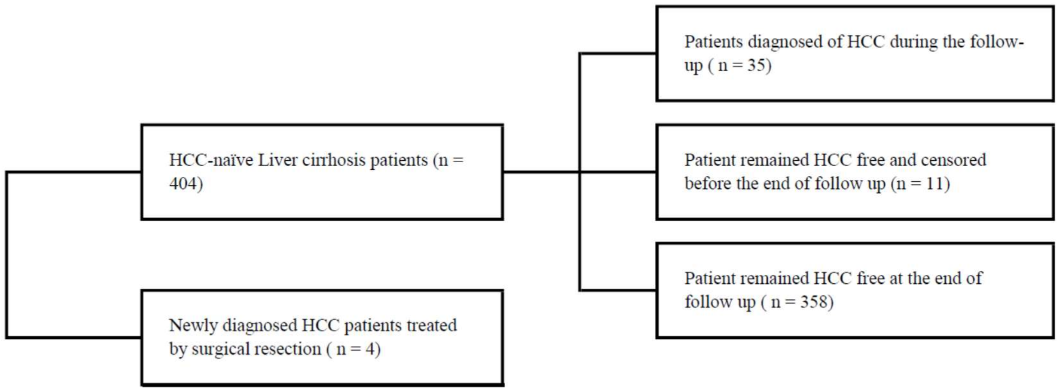

Supplementary Figure 1. The flow chart of this study.

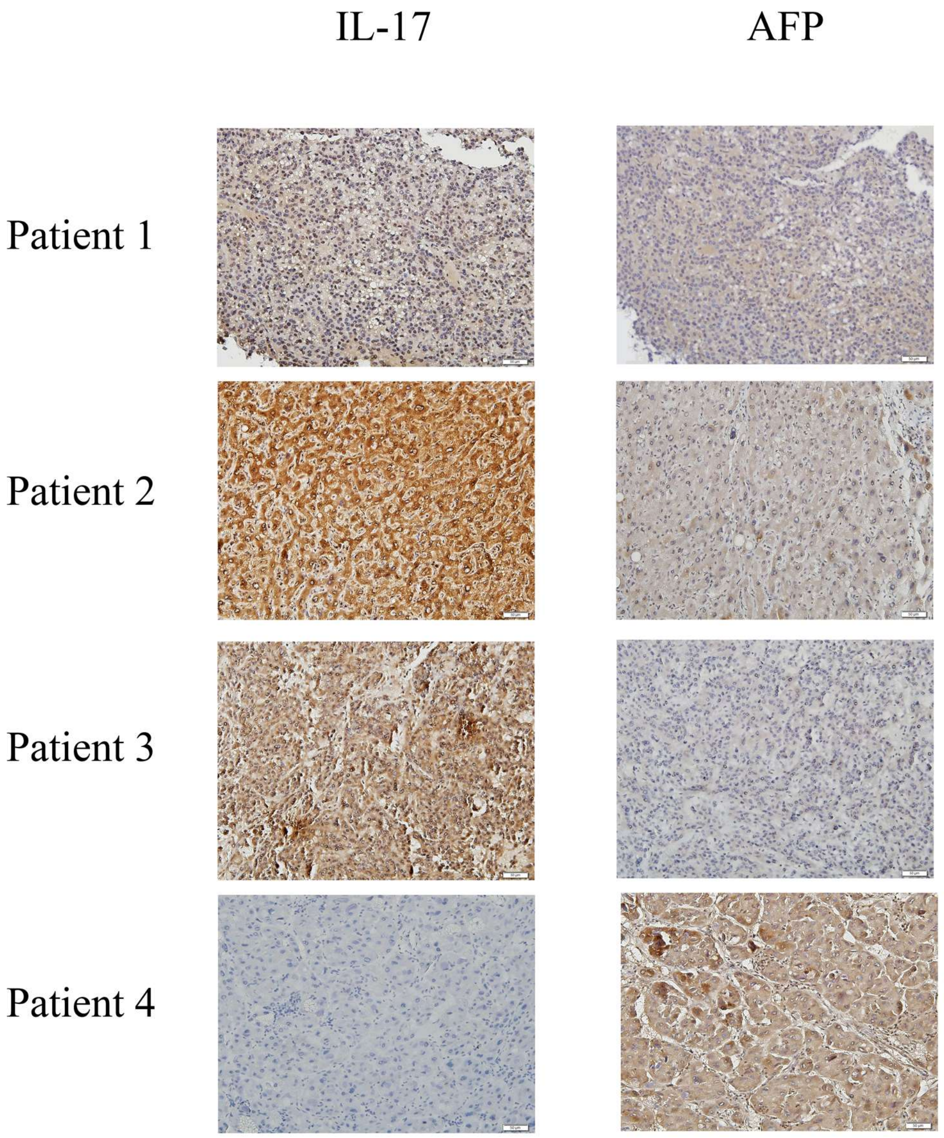

Supplementary Figure 2. The immunohistochemistry staining of IL-17 and AFP in tumor tissues of four hepatocellular carcinoma patients. Both IL-17 and AFP were detected in patients 1 and 2. IL-17 but not AFP were detected in patient 3. AFP but not IL-17 were detected in patient 4.
